# A Novel Technique for Managing Pancreaticojejunal Anastomotic Leak after Pancreaticoduodenectomy

**DOI:** 10.1155/2016/5392923

**Published:** 2016-06-14

**Authors:** Tufan Egeli, Tarkan Unek, Mucahit Ozbilgin, Mustafa Goztok, Ibrahim Astarcıoglu

**Affiliations:** HPB Surgery and Liver Transplantation Unit, Department of General Surgery, School of Medicine, Dokuz Eylul University, Inciralti, 35340 İzmir, Turkey

## Abstract

Pancreaticoduodenectomy (Whipple's procedure) remains the only definitive treatment option for tumors of the periampullary region. The most common and life-threatening complications following the procedure are pancreatic anastomotic leakage and subsequent fistula formation. When these complications occur, treatment strategy depends on the severity of anastomotic leakage, with patients with severe leakages requiring reoperation. The optimal surgical method used for reoperation is selected from among different options such as wide drainage, definitive demolition of the pancreaticojejunal anastomosis and performing a new one, or completion pancreatectomy. Here we present a novel, simple technique to manage severe pancreatic leakage via ligamentum teres hepatis patch.

## 1. Introduction

Pancreaticoduodenectomy (Whipple's procedure) has become a routine procedure for malignant periampullary tumors as well as benign disorders including chronic pancreatitis, diverticula, or large adenomas of the periampullary region. Resection itself is no longer problematic, although the reconstruction of pancreatic remnant is still challenging. Whereas mortality rate has been reduced to less than 5% in experienced centers, morbidity remains as high as 30 to 50% even in large series [1]. The most important complications related to pancreatic remnant are pancreaticojejunal anastomotic leakage (PJAL) and subsequent pancreatic fistula [1, 2]. In the case of severe PJAL, reoperation is crucial and it depends on the clinical status of a patient [3]. Pancreaticogastrostomy, repeat pancreaticojejunostomy, or total pancreatectomy may become necessary for its management. Here we present a simple and efficient technique using ligamentum teres hepatis (LTH) patch for the treatment of PJAL.

## 2. Case and Technique

**Figure 1:**
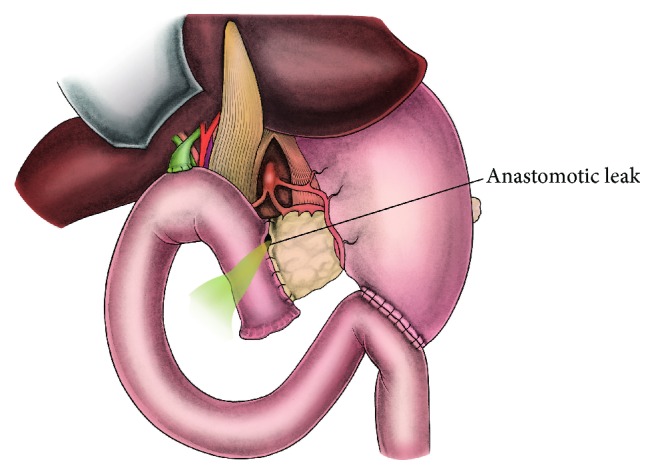
Anastomotic dehiscence and leakage from the right side of the pancreaticojejunal anastomosis.

**Figure 2:**
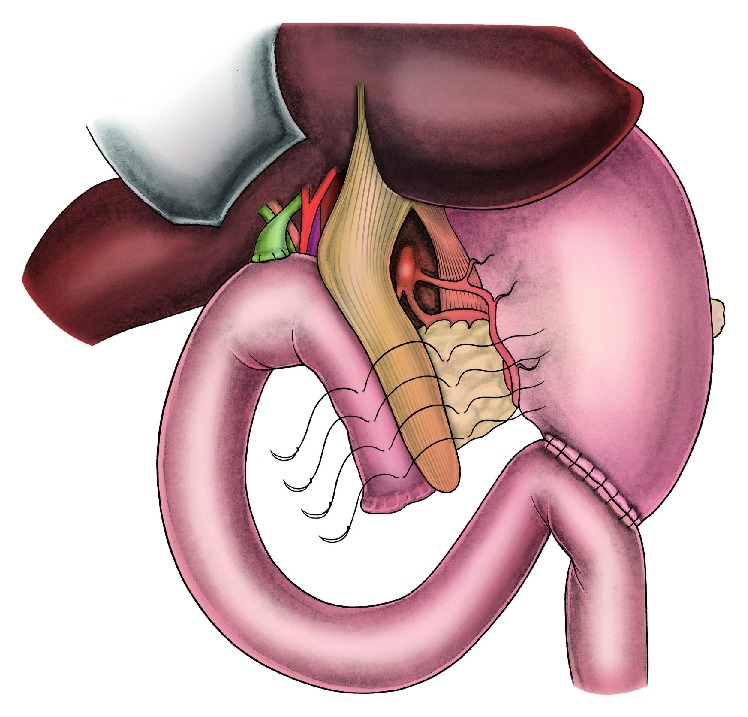
The mobilized ligamentum teres hepatis is placed onto the dehiscence side as a patch and fixed with interrupted sutures that were put on both pancreas and jejunum.

**Figure 3:**
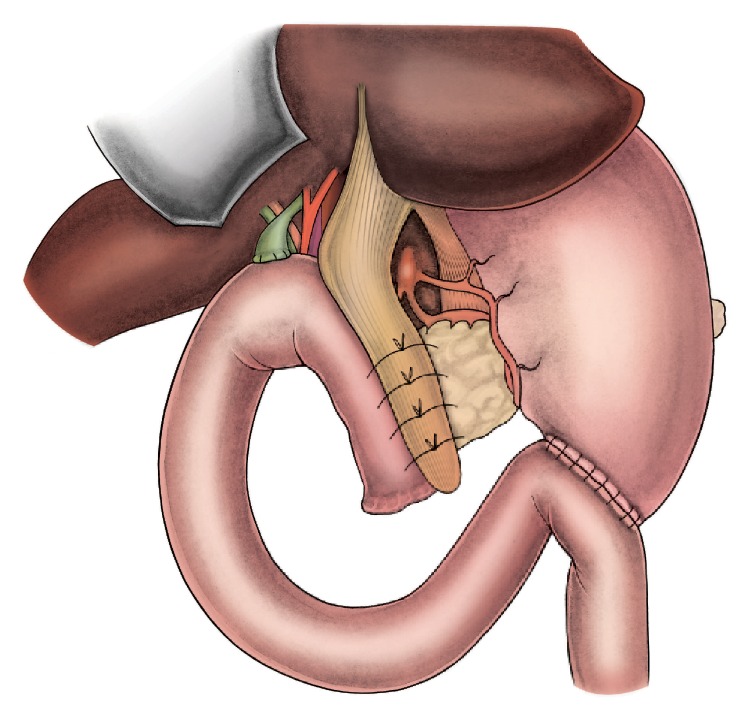
Gently ligated sutures serve to keep the teres hepatis patch in place.

A 72-year-old male patient underwent pancreaticoduodenectomy for carcinoma of pancreatic head in our clinic. During the operative evaluation, pancreatic texture was soft; the diameter of the Wirsung duct was 2 millimeters. After resection, we performed an end-to-side, duct-to-mucosa pancreaticojejunal anastomosis with 6/0 polyglactin sutures in order to manage the pancreatic remnant. We started intravenous somatostatin infusion (6 mg/24 hours) perioperatively to avoid subsequent pancreatic fistula formation. Histopathological examination revealed that the neoplastic mass was an adenocarcinoma (pT2N1). During the first 9 days after the surgery the patient was stable and there were no signs of PJAL. However, at day 10 postoperatively the patient developed continuous fever, hypotension, tachycardia, abdominal distention, signs of peritoneal irritation, and leukocytosis. The content of abdominal drainage was pure biliary. An abdominopelvic ultrasonography showed diffuse intra-abdominal fluid collection. All findings were consistent with abdominal sepsis secondary to severe PJAL; hence, we decided a reoperation. During the exploration at the surgery, the abdominal cavity was full of bile. After carrying out effective abdominal irrigation and debridement, we checked all anastomotic suture lines for leakage. A 4-millimeter dehiscence and leakage line was detected at the right side of the pancreaticojejunal anastomosis (Figure 1). Both the pancreatic remnant and jejunal limb tissue were observed to be inflammatory and edematous. Therefore, we deemed these tissues unsuitable for suturing securely, and we decided to use ligamentum teres hepatis (LTH) as a patch for reparation. Then we mobilized LTH and placed it onto the site of dehiscence and the upper part of the pancreaticojejunal anastomosis. LTH patch was fixed on both the pancreatic remnant and jejunal limb with interrupted polyglactin sutures, as shown in the illustrations (Figures 2 and 3). After achieving hemostasis and performing a sponge count, low negative pressure drains were placed in both peripancreatic areas. Finally, the pelvis and then the abdomen were closed. During the follow-up after surgery, no postoperative complications or signs of pancreatic leakage or fistula were observed. The patient was discharged with full recovery at day 9 postoperatively.

## 3. Discussion

Pancreaticojejunal anastomotic leak rate has been reported to occur in 6–24% of patients after pancreaticoduodenectomy [4–6], and it is associated with a mortality rate of 20% to 80% [7]. Conservative treatment usually yields excellent outcomes in mild PJAL [4–6]. However, in severe PJAL and particularly in the case of anastomotic dehiscence, reoperation becomes inevitable because it constitutes the primary cause of early postoperative mortality due to abdominal sepsis [3, 8]. In such cases, a surgeon should consider the extent of dehiscence, the clinical status of the patient, and the vitality of the pancreatic remnant to decide the best surgical management. Depending on the severity of leakage, simple wide drainage, creating a new pancreaticojejunal anastomosis, or even completion pancreatectomy can be the treatment of choice [1–3].

In the case of sufficient anastomosis but a small anastomotic leakage, irrigation of the leakage site and insertion of additional drains into the lesser sac is an appropriate approach. In the case of anastomotic disruption or subtotal dehiscence, however, surgical repair of the anastomosis should generally not be attempted because blood supply to that area is often not adequate and the inflammatory tissue may not be securely sutured [1, 9]. On the other hand, resecting a few centimeters of the pancreatic remnant and performing a new pancreaticojejunal anastomosis is a high-risk procedure with the possibility of a new anastomotic failure with persistent leakage and sepsis [2]. Some authors suggested salvage pancreaticogastrostomy as an alternative method to manage PJAL [10] but the experience with this technique seems limited in the literature. In cases with severe PJAL, completion pancreatectomy including splenectomy is still the only life-saving option, which should be performed before septic shock develops [11–14]. However, completion pancreatectomy should be limited to clinical circumstances in which there is no alternative because it is well known that more extensive interventions are hazardous and associated with higher morbidity and mortality [2–4].

The usage of mobilized ligamentum falciform hepatis for protection of gastroduodenal artery stump during pancreaticoduodenectomy has been defined previously [15]. In that method, the purpose is to minimize the risk for pseudoaneurysm formation at the site of the gastroduodenal artery stump in the event of pancreatic anastomotic leak. On the other hand, Hackert et al. described a successful technique of teres hepatis ligament patch as an additional supracapsular sealing structure for postoperative pancreatic fistula prevention after tumor enucleation from pancreas [16]. In a similar manner, Wu et al. reported usage of ligamentum teres hepatis as a patch to cover the pancreatic stump in order to avoid pancreatic fistula [17]. During the operation, we also recalled Graham's Technique for the treatment of perforated ulcers [18], and hence we decided to synthesize these techniques. So, we developed a novel method that was outlined above. Fortunately, it worked successfully and our patient was discharged with full recovery and without any complication. As a result, we suggest that this simple novel technique may be an alternative strategy as a less aggressive, organ preserving, and effective method when reoperation is necessary for PJAL after pancreaticoduodenectomy.
